# A nationwide population-based study in South Korea on a relationship between height and anosmia

**DOI:** 10.1038/s41598-021-86091-8

**Published:** 2021-03-24

**Authors:** Jeong Wook Kang, Young Chan Lee, Kyung Do Han, Kun Hee Lee

**Affiliations:** 1 Department of Otolaryngology-Head and Neck Surgery, Kyung Hee University School of Medicine, Seoul, Republic of Korea; 2 Department of Biostatistics, Catholic University College of Medicine, Seoul, Republic of Korea

**Keywords:** Olfactory system, Population screening

## Abstract

The relationship between anosmia and anthropometric factor has not been investigated sufficiently yet. Thus, the purpose of this study was to evaluate anthropometric risk factors of anosmia in an Asian population. Claims data of subjects over 20 years old who underwent a national health examination conducted by the Korean National Insurance Program between 2005 and 2008 were analyzed. They were followed up through the Korean National Insurance Service database. Individuals newly diagnosed with anosmia were identified after the initial health examination until the last follow-up date (December 31, 2016). The incidence of anosmia was high in females younger than 70 years old. The hazard ratio of anosmia was found to be higher in taller groups. The tallest quintile had higher risk than the shortest quintile (hazard ratio = 1.185, 95% confidence interval: 1.147–1.225) after adjusting for age, sex, BMI, income, smoking status, alcohol consumption, regular physical activity, hypertension, diabetes mellitus, and dyslipidemia. This study showed that the incidence of anosmia had a positive association with height. However, careful interpretation is needed to generalize our result because of the limitation of the study population. Further studies are needed to clarify the genetic or environmental causes of anosmia.

## Introduction

Anosmia means the loss of the sense of smell. It is also commonly used to indicate a decreased sense of smell. Prevalence of anosmia has diverse ranges according to study design. In previous studies, its prevalence has been estimated at 1.42 $\sim$ 24.5%^1^. The reason for such a wide range of its prevalence is due to the absence of a standardized diagnostic method to estimate the exact prevalence of anosmia^2^. Diagnostic tools for anosmia also differ from area to area.

The term anosmia is not a final diagnosis in general condition. The term is a kind of symptom caused by a series of condition. Causes of anosmia can be classified into conductive and sensory-neural ones according to etiology^3^. The most common cause of anosmia is upper respiratory tract infection (URI) that usually makes it difficult to transmit odorants^4^. Anosmia caused by URI usually could be resolved within several months. The shortness of its resolving period and conventional image of anosmia playing a less prominent role in its diagnosis have made people overlook the importance of anosmia^4,5^. However, when we look at the relationship between anosmia and neurodegenerative diseases such as dementia, anosmia cannot be overlooked. Anosmia is well known as an early sign of neurodegenerative disease^6–10^. Additionally, as quality of life (QOL) has gradually become a topic of well-being, several studies have reported that anosmia is related to QOL, especially in elderly individuals^11–13^.

Despite the clinical importance of anosmia, its risk factor in Asia has been rarely reported. Previous studies on non-Asian population have presented many risk factors of anosmia, including age, sex, air pollution, virus, smoking, income rate, diabetes mellitus, and neurodegenerative disease^13–17^. However, the relationship between anosmia and anthropometric factors has not been reported yet. Therefore, the objective of this study was to identify new anthropometric risk factor of anosmia in an Asian population through analyses of big database of Korean National Health Insurance Service (KNHIS).

## Results

### Demographics according to height quintile

We classified 9,937,806 subjects into five groups according to height. Supplementary Table 1 shows cutoff values for the classification. The number of participants in each group was about 2,000,000. Table 1 shows demographics of participants by height quintile. Among these five quintile groups, Q1 had lower alcohol consumption rate than Q5. This trend continuted from Q1 to Q5 (5.97%, 6.51%, 7.25%, 7.19%, 7.4%, respectively). The lowest income status ($<20\%$) showed a decreasing trend from Q1 to Q5 (23.92%, 21.21%, 20.02%, 19.66%, 18.95%). Q1 had higher incidence rate of hypertension and dyslipidemia than Q5. This trend continued from Q1 to Q5 for hypertension (26.7%, 25.23%, 26.62%, 25.25%, 24.96%, respectively) and dyslipidemia (18.94%, 18.34%, 18.7%, 18.07%, 17.26%, respectively). On the other hand, the incidence rate of chronic kidney disease (CKD) was reversed. It was increased with height (5.51%, 5.63%, 6.17%, 6.06%, 6.35%, respectively). Waist circumference (WC) showed an increasing trend from Q1 to Q5 (78.34 ± 8.65 cm, 79.41 ± 8.8 cm, 80.65 ± 8.84 cm, 80.84 ± 9.11 cm, 81.9 ± 9.49 cm). Glomerular filtration rate (GFR) showed a decreasing trend from Q1 ($89.49 \pm 39.05\,\hbox {mL/min}/{1.73}\hbox {m}^2$) to Q5 ($87.46 \pm 48.72\,\hbox {mL/min}./{1.73}\hbox {m}^2$). Other characteristics such as age, sex, smoking, fasting blood sugar (FBS), body mass index (BMI), blood pressure (BP), or triglyceride (TG) showed no obvious trends according to height, although they were significantly different among quintile groups.

**Table 1:** Demographics of subjects according to height quintile.

	Height				
	Q1	Q2	Q3	Q4	Q5
	(n = 2009895)	(n = 1966001)	(n = 1946883)	(n = 1966964)	(n = 2048063)
Age (y)	47.81±14.55	46.72±14.09	47.56±14.28	46.96±13.64	46.69±14
**Age group**					
20–39	611108 (30.4)	670227 (34.09)	621131 (31.9)	570379 (29)	644059 (31.45)
40–64	1106670 (55.06)	1046597 (53.23)	1045191 (53.69)	1160445 (59)	1155307 (56.41)
$\ge$65	292117 (14.53)	249177 (12.67)	280561 (14.41)	236140 (12.01)	248697 (12.14)
**Sex**					
Male	1079127 (53.69)	1068074 (54.33)	1145647 (58.85)	1069568 (54.38)	1089249 (53.18)
Female	930768 (46.31)	897927 (45.67)	801236 (41.15)	897396 (45.62)	958814 (46.82)
Smoking status (yes)	501221 (24.94)	515796 (26.24)	543057 (27.89)	515354 (26.2)	528511 (25.81)
**Alcohol consumption**					
Yes	119968 (5.97)	127939 (6.51)	141168 (7.25)	141390 (7.19)	151649 (7.4)
No	1889927 (94.03)	1838062 (93.49)	1805715 (92.75)	1825574 (92.81)	1896414 (92.6)
Lowest income status ($<20\%$)	480816 (23.92)	416938 (21.21)	389712 (20.02)	386767 (19.66)	388099 (18.95)
Hypertension	536868 (26.71)	496010 (25.23)	518344 (26.62)	496672 (25.25)	511100 (24.96)
Dyslipidemia	380656 (18.94)	360547 (18.34)	364066 (18.7)	355344 (18.07)	353566 (17.26)
CKD	110752 (5.51)	110775 (5.63)	120042 (6.17)	119247 (6.06)	130039 (6.35)
FBS level (mg/dL)	97.09±23.37	96.81±22.67	97.45±23.12	97.18±22.73	97.21±22.77
BMI (kg/$\hbox {m}^2$)	23.74±3.23	23.73±3.2	23.78±3.18	23.68±3.19	23.59±3.23
WC (cm)	78.34±8.65	79.41±8.8	80.65±8.84	80.84±9.11	81.9±9.49
**BP (mmHg)**					
Systolic	122.51±15.49	122.23±15.04	122.8±14.88	122.29±14.74	122.3±14.58
Diastolic	76.26±10.14	76.19±10.01	76.51±9.93	76.29±9.92	76.25±9.87
GFR (mL/min/$1.73\hbox {m}^2$)	89.49±39.05	88.85±42.7	88.06±44.69	87.77±44.56	87.46±48.72
$\log _{}{}$ TG	114.04 (113.95–114.13)	113.89 (113.8–113.98)	116.01 (115.92–116.11)	113.4 (113.31–113.49)	111.18 (111.09–111.27)

*CKD* chronic kidney disease, *FBS* fasting blood sugar, *BMI* body mass index, *WC* waist circumference, *BP* blood pressure, *GFR* glomerular filtration rate, *TG* triglyceride. Data are expressed as mean ± SD or n (%).

### Risk of anosmia according to age and sex

**Figure 1:**
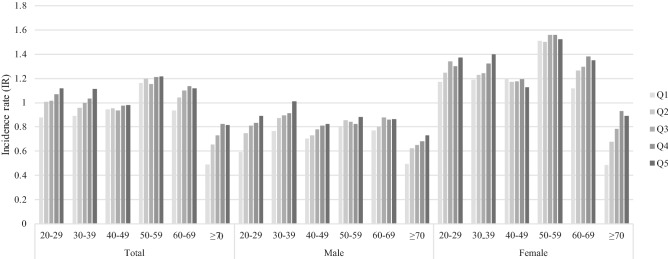
Unadjusted incidence rate of anosmia according to 10-year age group at baseline, sex and height (in quintiles).

The number of newly developed anosmia was 73,473 and the incidence rate was 1.019 (per 1000 person-years). Effects of age and sex on incidence of anosmia was analyzed in five groups (Q1–Q5) of height (Fig. 1). The incidence of anosmia was similar among age groups, excluding elderly individuals over 70 years who showed a low incidence rate. The elderly and male group showed relatively lower incidence rate than the younger and female groups. Peak incidence rate was shown in females in their 50s. The incidence of anosmia showed a trend of an increase with greater height in overall groups.

### Risk of anosmia according to height

**Figure 2:**
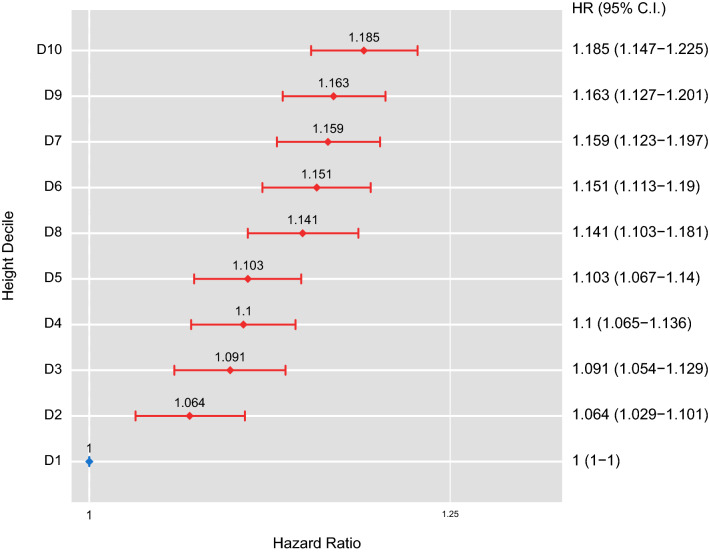
Hazard ratios (HRs) for anosmia by height decile after adjustment for age, sex, BMI, income, smoking status, alcohol consumption, regular physical activity, hypertension, diabetes mellitus, dyslipidemia. The Y-axes show deciles of height, while the X-axes show hazard ratios with reference to the 1st decile for height. Diamonds represent HRs, and horizontal lines indicate 95% confidence intervals.

Table 2 shows results of multivariate analysis between the risk of anosmia according to height. The Q5 height group had significantly higher risk of anosmia than the risk in the Q1 height group. The adjusted risk of anosmia was increased in the taller population. The model was adjusted for various risk factors such as age, sex, BMI, income, smoking, alcohol consumption, regular physical activity, hypertension, diabetes mellitus, and dyslipidemia. Risks of anosmia were higher in the under 40 age group, over 65 age group, taller males, and the low BMI group (< 18.5 kg/m$^2$). Figure 2 shows hazard (HR) and 95% confidence interval (CI) according to height deciles using the same adjusting model of Table 2. We used height deciles not quintiles in Table 2 because the modification of hazard ratio according to height was clearer in deciles. The high risk of anosmia in taller population means that taller individuals are more likely to have anosmia than shorter individuals.

**Table 2:** Multivariate Cox’s proportional hazard regression model of the relationship between height and risk of anosmia.

Group	Height				
	Q1	Q2	Q3	Q4	Q5
Total	1	1.063 (1.038–1.088)	1.092 (1.066–1.118)	1.116 (1.091–1.142)	1.137 (1.111–1.163)
**Age (y)**					
20–39	1	1.105 (1.059–1.153)	1.141 (1.093–1.191)	1.163 (1.113–1.214)	1.25 (1.199–1.303)
40–64	1	1.019 (0.988–1.05)	1.039 (1.007–1.071)	1.057 (1.026–1.089)	1.043 (1.012–1.074)
$\ge$65	1	1.126 (1.046–1.214)	1.217 (1.134–1.307)	1.296 (1.205–1.393)	1.281 (1.192–1.377)
**Sex**					
Male	1	1.096 (1.057–1.137)	1.13 (1.091–1.171)	1.135 (1.095–1.176)	1.207 (1.165–1.251)
Female	1	1.038 (1.007–1.071)	1.063 (1.03–1.097)	1.101 (1.068–1.135)	1.085 (1.053–1.118)
**BMI** (kg/m^2^)					
<18.5	1	1.041 (0.916–1.184)	1.131 (0.993–1.288)	1.203 (1.063–1.361)	1.333 (1.185–1.499)
18.5–23	1	1.07 (1.03–1.112)	1.133 (1.09–1.177)	1.145 (1.103–1.188)	1.18 (1.137–1.224)
23–25	1	1.065 (1.017–1.116)	1.094 (1.045–1.146)	1.125 (1.074–1.178)	1.098 (1.048–1.149)
25–30	1	1.05 (1.005–1.096)	1.038 (0.994–1.084)	1.061 (1.016–1.108)	1.088 (1.042–1.135)
$\ge$30	1	1.061 (0.938–1.199)	1.019 (0.899–1.155)	1.081 (0.957–1.222)	1.105 (0.978–1.248)

Model: adjusted for age, sex, BMI, income, smoking status, alcohol consumption, regular physical activity, hypertension, diabetes mellitus, dyslipidemia.

*BMI* body mass index, *HR* hazard ratio, *95% CI* 95% confidential interval of hazard ratio. Data are expressed as HR (95% CI).

## Discussion

There are a series of studies to show a correlation between olfactory impairment and anthropometric measures. Among anthropometric measures, BMI was the most focused characteristic. Some studies showed clues of a negative correlation between olfactory function and BMI. A recent National Health and Nutrition Examination Survey based study reported that olfactory dysfunction has a relationship with high BMI and WC in middle-aged women^18^. Another previous study also showed a similar result in male subjects^19^. Anatomically, over-weight could be correlated with low olfactory bulb volume^20^. On the other hand, normal olfactory function may prevent weight loss by keeping a flavor of taste, especially in elderly people. Lowering of the sense of smell is related to nutritional risk^21^. Decreased olfactory function was associated with underweight^22^. A nutriepigenomic study using 474 subjects reported an interesting result showing genomic links between olfactory functions and anthropometric measures^23^. In their study, methylation levels of olfactory pathway genes were associated with BMI and WC.

In this study, the authors used a single diagnostic code (R430) standing for anosmia. Research using non-fatal disease codes such as anosmia (R430) obviously has limitations. Because such diagnostic codes are easily omitted in a general situation. However, conversely, the authors thought that if such minor diagnostic code is registered in claims data, we can expect the code was the only symptom code, or it can be a relatively chief complaint in a participant. The authors intended to detect anthropometric risk factors in the participant having anosmia as a relatively major symptom. For the same purpose, this study did not use a combined code, ’impairment of the sense of smell and taste’. The authors expected that using a single minor diagnostic code may reduce the impact of confounding factors.

This study revealed that incidence of anosmia was related to greater height. It means that anthropometric factors such as height could affect the development of anosmia. Anthropometric features of individuals are determined by genetic and environmental factors. Despite bimodal influence, the effect of genetic factors on height is greater than that of environmental factors. Genetic influence on height has reached 60–80%^24–26^. The percentage of genetic influence is very constant regardless of populations, although different populations have different mean heights^27^. The genetic influence on height has also been found in South Korean children^28^. From this point of view, anosmia could be affected by some genetic factors.

This study revealed that greater height was significantly associated with higher risk of anosmia. This study chose a model after adjusting for confounding factors such as age, sex, BMI, income, smoking, alcohol consumption, regular physical activity, hypertension, diabetes mellitus, and dyslipidemia. Although this study failed to show age-related increase in incidence rate of anosmia as shown in Fig. 1, the fact that smell identification ability had a tendency to decrease with age was well established. Therefore, we included age in our model in Table 2 and Fig. 2. In this study, the prevalence of dyslipidemia and hypertension was greater in the shorter height group. It is well known that hypertension and dyslipidemia are related to family history. Patient who have a family history of hypertension would have a two to fourfold higher risk of getting the disease^29,30^. Family history of dyslipidemia is also associated with the presence of dyslipidemia in pediatrics^31–33^. The relationship between human height and these two diseases with a family history could be explained by the influence of genetic and environmental factors.

This report is meaningful to reveal that the height is related to the risk of anosmia. A major features of this study is large subject size. This study could involve all claims in South Korea because we used a big database of the Korean National Health Insurance (NHI) and the Korean Medical Aid programs. The database contains claims over 50 million which is almost 99% of the Korean population^34^. The database also contains information about clinical diagnosis including anosmia. The information was collected from all medical institutions that requested claim to the KNHIS in South Korea. Another advantage of this study was the homogeneity of the study population which consisted of South Korean. The homogeneity could reduce bias induced by racial heterogeneity^35^.

There are a kinds of limitations in this study. First, there could be some undetected patients who didn’t visit a hospital despite their symptoms. Second, there is no definite diagnostic guideline for anosmia in South Korea. Thus, some clinics would diagnose anosmia without any subjective or objective diagnostic processes. Third, anosmia is a symptom that could have multiple causes. The most common cause is upper respiratory tract infection. Therefore, it could be unreasonable to interpret the results of this study under a hypothesis that anosmia is a homogeneous disease. Fourth, this study did not consider environmental factors such as climate or dwelling place. Fifth, the population enrolled in this study did not cover all patients with anosmia. The national health examination enrolled approximately one-fourth (10 million) of the total population over 20 years (39 million). Sixth, we did not consider the population unmet for medical care. The number of those unmet for medical care is about 9% of South Korean^36^. They tend to have low economic status. If their incidence was reflected in this study, the incidence would be higher. Therefore, despite the huge number of the study population, it is unclear whether the subjects of this study could represent the whole Korean or Asian population.

In several studies, anthropometric measures such as BMI and WC have been used as predictors of risks of other diseases^37,38^. Despite the purpose of this study was to analyze the correlation between anthropometric factors and anosmia, but failure to prove the relationship between BMI and WC is another limitation. Authors think a further study should be conducted to reveal the relationship. However, as shown in Table 1, according to the height quantile, BMI does not have a tendency, but WC tends to increase as the height increases, so this should be considered in the additional research.

A recent study by Roh et al. mentioned the relationship between coronary artery disease and anosmia in South Korea^39^. However, this study was conducted before the study of Roh et al.. Moreover, in our preliminary study, we could limitedly found that ischemic heart disease (IHD) associated with anosmia, but myocardial infarction (MI) didn’t. The authors could not assurance this comorbidity could be a confounding factor. This limitation about confounding factors should be considered in the future study. On the other hand, as anosmia is a type of neurologic symptom, we concerned that the validation power would be degraded if we consider neurodegenerative diseases as confounding factors. Therefore, in this study, the diseases such as Alzheimer’s disease, Parkinson’s disease, and stroke were not included in confounding factors.

In summary, results of this study suggest that anosmia is associate with greater height as one of its anthropometric factors. Further studies are needed to reveal the mechanism of how height affects the risk of anosmia.

## Methods

The methods for Lee et al. study was grossly referenced in this study^40^.

### Study design and data sources

This is a large-scale retrospective cohort study. This study analyzed two kinds of databases which are the ‘KNHIS claims database’ and the ‘Korean Health Examination database’. The Korean government offers the health examination service to a person every year or every other year according to their occupations. General Koreans should undergo the health examination for office workers, heads of household, and household members over 40 years old. Therefore, the ’Korean Health Examination database’ contains extensive health information of Koreans. The health information includes not only disease information, but also anthropometric measurements. We used the ‘Korean Health Examination database’ to make groups of height. At the same time, we used the ’KNHIS claims database’ to evaluate the occurence of anosmia of the same subject. The Korean NHI database is a valuable dataset for large population epidemiologic studies^41^. The Korean NHI program is a basic health care system of South Korea serviced for all Korean. Therefore, the KNHIS claims database includes whole claims data from the Korean Medical Aid program, the Korean NHI program, and other medical insurance programs from 2009 to 2016. The database covers most Korean population. For example, the database included 48,341,311 people in 2006 and 51,574,044 people in 2015. The database was built up with the International Classification of Disease, Tenth Revision, Clinical Modification (ICD-10-CM) codes. The authors analyzed the database from 2009 to 2016.

### Study population and definition of anosmia

**Figure 3:**
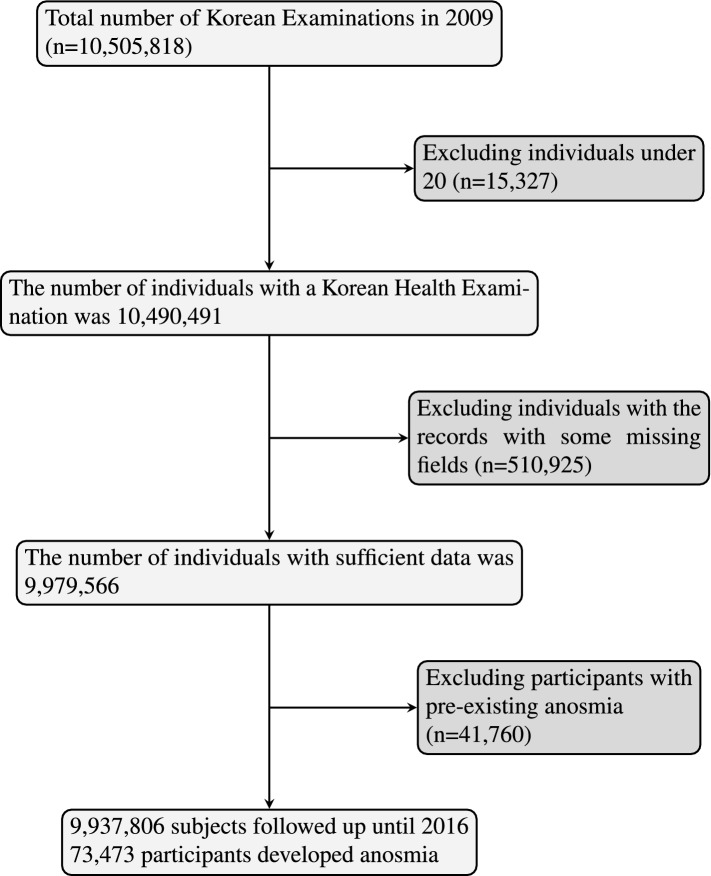
A flow chart of the study.

This study used the Korean Health examination database including anthropometric measures, which made us to evaluate the incidence of anosmia in South Korea. First, a customized data was extracted from the Korean Health examination database to use the health examination data. We also requested individual age, sex, anthropometric features, underlying diseases (hypertension, diabetes mellitus, and dyslipidemia), and behavioral features (smoking habit, alcohol consumption, and regular physical activity). The total number of health examinations in Korean adults over 20 years of age was 10,490,491 in 2009. If individuals had missing records (n = 510,925) or history of anosmia (n = 41,760) in the first health examination, they were excluded from the analysis to avoid confounding effect. Ultimately, the study population consisted of 9,937,806 subjects. The date when the people underwent their first health examination was defined as an initial date to start following up whether they newly are diagnosed with anosmia. After that, we followed up newly diagnosed anosmia cases which recorded as R430 in KNHIS claims database during the study period (ICD-10-CM code: R430). All study population was reviewed from the initial date to the last follow-up date of study design (December 31, 2016) (Fig. 3). No need to acquire institutional review board approval was confirmed by the Institutional Review Board of Kyung Hee University Hospital at Gangdong because this study didn’t use this identifiable individual information. All research was performed according to study guidelines and regulations.

### Measurements

Age, sex, and other anthropometric features such as height and weight were basically checked and analyzed in the process of the Korean health examination. The anthropometric measurements were tested under light clothing status. BMI was measured by how weight (kg, kilogram) divided by the square of height ($\hbox {m}^2$, meter). Blood pressure (BP) was measured under sitting position after short resting over five minutes. Alcohol consumption, smoking habit and income rate were evaluated using questionnaires. Serum glucose and cholesterol levels were measured using blood samples taken after overnight fasting. The national health examinations were permitted to some hospitals. The hospitals should undergo regular quality control to get certification from NHI service.

### Definition of chronic diseases

Subjects who have chronic diseases such as hypertension and diabetes mellitus may take medications. The medications could affect anosmia development. Therefore, adjustments for comorbidities were needed in the analysis. We used the Korean NHI Service database to identify comorbidities of subjects. The presence of diabetes was defined as: (1) at least one claim per year for a prescription of antidiabetic medication under ICD-10 codes E10–14, or (2) fasting glucose level $\ge$ 110 mg/dL^40^. The presence of hypertension was defined as: (1) at least one claim per year for the prescription of an antihypertensive agent under ICD-10 codes I10–I15, or (2) systolic/diastolic BP $\ge$ 140/90 mmHg^40^. The presence of dyslipidemia was defined as: (1) at least one claim per year for the prescription of an antihyperlipidemic agent under ICD-10 code E78, or (2) total cholesterol $\ge$ 239 mg/dL^40^. We verified the test values in the health examination database.

### Statistical analysis

All subjects were classified by height specific quintiles in each age and sex group (Supplementary Table 1). The lowest 20% income population was excluded. Sex and age are well-known risk factors of anosmia^13,17^. Thus, we described the incidence of anosmia according to age and sex. Because it is unclear which anthropometric features affect anosmia, we broadly accessed multiple confounding factors. We finally chose an adjusted model including confounding factors as many as possible. The selected model included body mass index (BMI) that was revealed as a confounding factor of anosmia incidence in a previous study^42^. The incidence rate of anosmia might be associated with smoking habit, alcohol consumption, regular physical activity, and individual income level. We regarded hypertension, diabetes mellitus, and dyslipidemia as confounders though these diseases weren’t definitely demonstrated as confounders of anosmia. These confounders were included in our adjusted analyses. When we analyzed the association between risk of anosmia and individual height, we applied Cox’s proportional hazards regression models. We presented each height group hazard ratio (HR) and 95% confidence interval (CI) relative to the lowest quintile. We also presented HR and 95% CI of each height deciles by Cox’s proportional hazards regression after adjusting those confounding factors. We applied the analyses in each group divided by sex and age (<40, 40-64 , and $\ge$65 years old). We used a log-log cumulative survival graph and time-dependent variable Cox model to check proportional hazard assumptions. SAS software (ver. 9.4; SAS Institute, Cary, NC, USA) and R project for Statistical Computing ver. 3.5.3 (http://www.r-project.org) were used for statistical analyses in this study.

The Institutional Review Board (IRB) of Kyung Hee University Hospital at Gangdong approved this study. The IRB waived informed consent requirement because this study didn’t have a plan to access identifiable information of subjects. All researchers conducted this study according to relevant guidelines and regulations.

## Supplementary Information

Supplementary Information.

## References

[1] Nordin S, Bramerson A (2008). Complaints of olfactory disorders: Epidemiology, assessment and clinical implications. Curr. Opin. Allergy Clin. Immunol..

[2] Dhong HJ, Shin DB, Rho HI, Chung SK, Chu KC (2001). Clinical analysis of olfactory disorders. Korean J. Otorhinolaryngol. Head Neck Surg..

[3] Holbrook EH, Leopold DA (2006). An updated review of clinical olfaction. Curr. Opin. Otolaryngol. Head Neck Surg..

[4] Temmel AF (2002). Characteristics of olfactory disorders in relation to major causes of olfactory loss. Arch. Otolaryngol. Head Neck Surg..

[5] Hummel T, Nordin S (2005). Olfactory disorders and their consequences for quality of life. Acta Otolaryngol..

[6] Graves AB (1999). Impaired olfaction as a marker for cognitive decline: Interaction with apolipoprotein e epsilon4 status. Neurology.

[7] Eichenbaum, H., Morton, T. H., Potter, H. & Corkin, S. Selective olfactory deficits in case hm. *Brain* **106 (Pt 2)**, 459–72 (1983). 10.1093/brain/106.2.459 6850278

[8] Huff FJ (1987). The neurologic examination in patients with probable alzheimer’s disease. Arch. Neurol..

[9] Serby M (1996). Olfactory identification deficits in relatives of alzheimer’s disease patients. Biol. Psychiatry.

[10] Nordin S, Murphy C (1996). Impaired sensory and cognitive olfactory function in questionable alzheimer’s disease. Neuropsychology.

[11] Deems DA (1991). Smell and taste disorders, a study of 750 patients from the university of pennsylvania smell and taste center. Arch. Otolaryngol. Head Neck Surg..

[12] Bromley, S. M. Smell and taste disorders: a primary care approach. *Am Fam Physician* **61**, 427–36, 438 (2000). 10670508

[13] Doty RL, Kamath V (2014). The influences of age on olfaction: A review. Front. Psychol..

[14] Ruan Y, Zheng XY, Zhang HL, Zhu W, Zhu J (2012). Olfactory dysfunctions in neurodegenerative disorders. J. Neurosci. Res..

[15] Doty RL (2017). Olfactory dysfunction in neurodegenerative diseases: is there a common pathological substrate?. Lancet Neurol..

[16] Bramerson A, Johansson L, Ek L, Nordin S, Bende M (2004). Prevalence of olfactory dysfunction: The skovde population-based study. Laryngoscope.

[17] Doty RL (1984). Smell identification ability: Changes with age. Science.

[18] Gallo, S. *et al.* Associations of olfactory dysfunction with anthropometric and cardiometabolic measures: Findings from the 2013–2014 national health and nutrition examination survey (NHANES). *Physiol. Behav.* **215**, (2020). 10.1016/j.physbeh.2019.112702 31629766

[19] Griep MI (1997). Odour perception in relation to age, general health, anthropometry and dental state. Arch. Gerontol. Geriatr..

[20] Poessel, M. *et al.* Reduced olfactory bulb volume in obesity and its relation to metabolic health status. *Front. Hum. Neurosci.* **14**, (2020). 10.3389/fnhum.2020.586998 PMC7729134 33328935

[21] Schiffman SS, Warwick ZS (1993). Effect of flavor enhancement of foods for the elderly on nutritional status: Food intake, biochemical indices, and anthropometric measures. Physiol. Behav..

[22] Fluitman KS (2019). The association of olfactory function with BMI, appetite, and prospective weight change in Dutch community-dwelling older adults. J. Nutr. Health Aging.

[23] MENA project *et al.* Associations between olfactory pathway gene methylation marks, obesity features and dietary intakes. *Genes Nutr.* **14**, 11 (2019). 10.1186/s12263-019-0635-9 PMC6485100 31057674

[24] Silventoinen K (2003). Determinants of variation in adult body height. J. Biosoc. Sci..

[25] Harris JR, Magnus P, Tambs K (2002). The norwegian institute of public health twin panel: A description of the sample and program of research. Twin Res..

[26] Stunkard AJ, Foch TT, Hrubec Z (1986). A twin study of human obesity. JAMA.

[27] Silventoinen K (2003). Heritability of adult body height: A comparative study of twin cohorts in eight countries. Twin Res..

[28] Lee EM, Park MJ, Ahn HS, Lee SM (2012). Differences in dietary intakes between normal and short stature korean children visiting a growth clinic. Clin. Nutr. Res..

[29] Muldoon, M. F., Terrell, D. F., Bunker, C. H. & Manuck, S. B. Family history studies in hypertension research. review of the literature. *Am. J. Hypertens.* **6**, 76–88 (1993). 10.1093/ajh/6.1.76 8427666

[30] Winnicki M (2006). Lifestyle, family history and progression of hypertension. J. Hypertens..

[31] Filgueiras MS, Vieira SA, Ribeiro AQ, Novaes JF (2019). Family history is associated with the presence of dyslipidemia in pre-school children. Rev. Paul Pediatr..

[32] Kelishadi R (2014). Genetic association with low concentrations of high density lipoprotein-cholesterol in a pediatric population of the middle east and north africa: the caspian-iii study. Atherosclerosis.

[33] Yamada Y (2007). Prediction of genetic risk for dyslipidemia. Genomics.

[34] Cheol Seong, S. *et al.* Data resource profile: The national health information database of the national health insurance service in south korea. *Int. J. Epidemiol.* **46**, 799–800 (2017). 10.1093/ije/dyw253 PMC5837262 27794523

[35] Savada, A. M., Shaw, W., Library of, C. & Federal Research, D. *South Korea : a country study* (Federal Research Division, Library of Congress : For sale by the Supt. of Docs., U.S. G.P.O., Washington, D.C., 1992).

[36] Kweon S (2014). Data resource profile: the korea national health and nutrition examination survey (knhanes). Int. J. Epidemiol..

[37] Qu Y (2020). Association of body mass index with risk of cognitive impairment and dementia: A systematic review and meta-analysis of prospective studies. Neurosci. Biobehav. Rev..

[38] Lee, C. M. *et al.* Association of anthropometry and weight change with risk of dementia and its major subtypes: A meta-analysis consisting 2.8 million adults with 57 294 cases of dementia. *Obesity Reviews: An Official Journal of the International Association for the Study of Obesity* **21**, e12989 (2020). 10.1111/obr.12989 PMC7079047 31898862

[39] Roh D (2021). The association between olfactory dysfunction and cardiovascular disease and its risk factors in middle-aged and older adults. Sci. Rep..

[40] Lee, Y. B. *et al.* Association between Height and Actinic Keratosis: A Nationwide Population-based Study in South Korea. *Sci. Rep.* **8**, (2018). 10.1038/s41598-018-29155-6 PMC6052058 30022092

[41] Song SO (2014). Background and data configuration process of a nationwide population-based study using the korean national health insurance system. Diabetes Metab. J..

[42] Patel, Z. M., DelGaudio, J. M. & Wise, S. K. Higher body mass index is associated with subjective olfactory dysfunction. *Behav. Neurol.* **2015**, (2015). 10.1155/2015/675635 PMC4496469 26199458

