# Supplementary material for: A nationwide population-based study in South Korea on a relationship between height and anosmia
Source: Sci Rep. 2021 Mar 24;11:6753. doi: 10.1038/s41598-021-86091-8 (PMC7991667; doi:10.1038/s41598-021-86091-8)
Supplement: Supplementary file 1 — Supplementary Information. [file 41598_2021_86091_MOESM1_ESM.doc]

Original article

**A Nationwide Population-based Study in South Korea on a Relationship between Height and Anosmia**

Jeong Wook Kang^1^, Young Chan Lee^1^, Kyungdo Han^2^, and Kun Hee Lee^1*^.

^1^Department of Otorhinolaryngology‐Head and Neck Surgery, Kyung Hee University School of Medicine, Seoul, Korea

^2^Department of Biostatistics, College of Medicine, The Catholic University of Korea, Seoul, Korea

Correspondence and reprint requests should be addressed to: Kun Hee Lee MD, Ph.D., Department of Otorhinolaryngology‐Head and Neck Surgery, Kyung Hee University School of Medicine, Kyung Hee University Hospital at Gangdong, 892, Dongnam-ro, Gangdong-gu, Seoul, Republic of Korea, e-mail: nose4u@gmail.com

Supplementary table 1. Table that illustrates the height of subjects according to age, sex and.height quintile (cm).

| **Height** |  |  |  |  |  |  |  |  |
| --- | --- | --- | --- | --- | --- | --- | --- | --- |
| **Age (years)** | SEX | number of individuals | Minimum | 1^st^ quintile | 2^nd^ quintile | 3^rd^ quintile | 4^th^ quintile | Maximum |
| **20-29** | male | 643532 | 160 | 169 | 173 | 175 | 179 | 208 |
|  | female | 583307 | 149 | 157 | 160 | 163 | 166 | 198 |
| **30-39** | male | 1376379 | 159 | 168 | 171 | 174 | 178 | 207 |
|  | female | 513686 | 148 | 156 | 159 | 161 | 164 | 197 |
| **40-49** | male | 1388953 | 157 | 166 | 169 | 172 | 175 | 202 |
|  | female | 1214315 | 145 | 153 | 156 | 159 | 162 | 199 |
| **50-59** | male | 1046680 | 154 | 163 | 167 | 169 | 173 | 199 |
|  | female | 1065975 | 143 | 151 | 154 | 157 | 160 | 204 |
| **60-69** | male | 660030 | 152 | 161 | 165 | 167 | 171 | 198 |
|  | female | 693874 | 140 | 149 | 152 | 155 | 158 | 198 |
| **>70** | male | 336091 | 159 | 159 | 163 | 165 | 169 | 199 |
|  | female | 414984 | 135 | 145 | 148 | 151 | 154 | 197 |
